# Encouraging death communication in a death-avoidant society: analysis of interviews with death café organizers

**DOI:** 10.1186/s12913-023-09967-7

**Published:** 2023-09-04

**Authors:** Kae Ito, Shuji Tsuda, Mayumi Hagiwara, Tsuyoshi Okamura

**Affiliations:** 1 Tokyo Metropolitan Institute for Geriatrics and Gerontology, Research Team for Human Care, Tokyo, Japan; 2 Graduate School of Kagawa Nutrition University, Sakado, Japan; 3 Tokyo Metropolitan Institute for Geriatrics and Gerontology, Research Team for Promoting Independence and Mental Health, Tokyo, Japan

**Keywords:** Death café, Death and dying, Death communication, Community

## Abstract

**Background:**

Post-war Japanese tend to avoid discussion of death, resulting in a lack of death communication within clinical settings. However, with the aging of society, the significance of conversations and decisions related to end-of-life issues has grown. In 2007, the government established guidelines for decision-making in end-of-life care. Nonetheless, death communication remains a challenge for healthcare professionals in clinical settings.

In contrast, death cafés have spontaneously emerged within communities as informal gatherings to openly discuss and explore death. Learning from death café organizers may help healthcare professionals encourage death communication in a death-avoidant society.

Therefore, a qualitative study was conducted to describe death cafés by examining the underlying motivation and practices through interviews with death café organizers.

**Methods:**

Individual in-depth interviews were conducted with death café organizers. Two key aspects were explored: 1) the underlying motivations of organizers; and 2) the practices and challenges encountered in running death cafés. The interviews were transcribed verbatim and analyzed using a qualitative descriptive approach. Thematic analysis was used.

**Results:**

The study identified two themes representing the underlying motivation of death café organizers: individually-oriented and community-oriented. These themes exhibited contrasting orientations and were collectively termed “individual-community orientation”.

Regarding the practice of death cafés, the focus was on the “attitude towards having attendees with and without grief in the same session.” Participants’ attitudes towards this aspect fell into two categories with opposing orientations: “purification” and “inclusion.” The “purification-inclusion orientation” was more prevalent among organizers who initiated death cafés due to their personal experiences.

A matrix was created to categorize death cafés based on their underlying motivations (individual vs. community-oriented) and practices (purification vs. inclusion). This classification resulted in quadrant 1 (community-oriented, inclusive) and quadrant 3 (individually-oriented, purification). Notably, death cafés in quadrant 1 were often held in temples.

**Conclusions:**

Japanese death cafés can be classified into two categories: individually and purification-oriented and community and inclusive-oriented categories. Healthcare professionals can learn valuable insights from death café organizers, particularly in promoting death communication. Specifically, temple death cafés, with their inclusive practices and orientation towards community, can be particularly beneficial in fostering inclusivity and community engagement.

## Background

Post-war Japanese tend to avoid discussion of death. According to Becker [1], postwar Japanese society lacked religious and death-related education, and the topic of death was often regarded as polluted or shameful. However, around the 1970s, Japan’s population began to age rapidly [2], leading to an increased importance of discussions and decisions related to end-of-life issues. In 1992, the Japan Medical Association’s Council on Bioethics stated that death was a taboo subject in Japan, and that there was insufficient discussion about end-of-life care, but that with the aging of the population, the issue of end-of-life care had become a pressing problem that could not be ignored [3]. The Japan Medical Association’s Council on Bioethics acknowledged that conventional paternalistic medicine cannot adequately address this problem, and it recognized the need for a new way of thinking that includes patient self-determination [3]. Subsequent discussions emphasized the importance of respecting the individual’s self-determination, leading to the issuance of the first guidelines for decision-making in end-of-life care in 2007 [4], which underscored the significance of involving patients in these decisions. In short, from the standpoint of clinicians working at hospitals, Japanese society is gradually moving in the direction of encouraging people to think about their own death and dying.

End-of-life care conversations include talking about prognosis, preference, and priorities, as well as hopes and fears regarding death and dying [5]. In the reality of the clinical setting, death communication is often a challenge for healthcare professionals [6]. It has been suggested that, though palliative care holds value throughout the disease trajectory, some of the most difficult conversations in palliative care involve end-of-life issues [5]. Similarly, code status discussions, which are associated with a decrease in invasive procedures for terminally ill patients, have shown that approximately half of the patients with advanced-stage diseases were not engaged in these discussions on admission [7]. Various barriers exist when it comes to communicating about death. These barriers include patients’ unreadiness [5], and physicians’ concerns about a negative impact on patients’ or caregivers’ emotional well-being [8].

In contrast, outside of the clinical setting, death cafés have spontaneously emerged within communities as informal gatherings where individuals come together to discuss and explore the topic of death. These community-led gatherings are not necessarily led by professionals involved in end-of-life care, but they serve as a space for open discussions about death. The question comes to mind for us healthcare professionals who are struggling to initiate death communication: how is death communication conducted in death cafés?

### Death café context -Underwood’s “Death Cafe”

The definitions of “Death Cafe” and “death café” are as follows. When referring to death cafés in general, the term “death café” is used. When referring to the Underwood-specific Death Cafe, such as Underwood’s Death Cafe running guide, or, registered on an Underwood-operated website, and licensed to use the title “Death Cafe”, the term “Death Cafe” is used.

The roots of death cafés can be traced back to 1999 when Swiss sociologist Bernard Crettaz held a café mortel to discuss death after the death of his wife [9]. Hammer et al. [10] presented the sentiments of Crettaz in her paper—“a death-defiant/ avoidant culture suffers the ill effect of death’s ‘tyrannical secrecy’”. Inspired by Crettaz’s work, British social entrepreneur John Underwood organized a death café in London in 2011. Underwood codified the concept of death café [11] and established a central website where people who agreed to abide by the principles he set forth could use the name “Death Cafe” for their events and announce them on the site.

A Death Cafe as defined by Underwood is a group-directed discussion of death without any specific agenda, topics, questions, or speakers. Its objective is to increase awareness of death, with the aim of helping people make the most of their finite lives [11]. The movement is driven by volunteers who are committed to creating a safe space for people to discuss death. The principles of Death Cafe, as set forth by Underwood, state that it is always offered with no intention of leading people to any conclusion, product, or course of action. It is an open, respectful, and confidential space where people can express their views about life and death safely. The events are held on a not-for-profit basis and are accompanied by refreshments [12].

Since the publication of Underwood’s Death Cafe running guide [12], this movement has spread rapidly across Europe, North America, and Australia, with over 16,000 death cafés registered as Death Cafes on the central website operated by Underwood and held in 85 countries since September 2011 (as of July 14, 2023) [13]. These cafés provide a space where grief, loss, and fear, which may not be warmly welcomed in other settings, are greeted with compassion and understanding [14]. The widespread adoption of death cafés in such a short time reflects the growing need for more open and flat conversations about death, and highlights the lack of such spaces in current society [15].

### Japanese death café history

The first event using the name “death café” in Japan was held around 2010 [16]. Death cafés that refer strictly to Underwood’s Death Cafe running guide spread gradually throughout the country [16]. Since around 2016, newer and more flexible forms of death cafés have emerged, such as those combined with picture book readings, themed workshops, and talks by professionals. Professionals were invited from diverse fields, for example, gerontologists, Buddhist monks, and service providers cleaning a messy house where older people live [16]. Richards [15] reported that local adaptations of death cafés, for example, addressing specific themes in advance, inviting professionals to give talks, incorporating mindfulness practices at the beginning or end, using prompt cards, and even organizing poetry readings, have been observed internationally. They found that the reason for these adaptations was simply because organizers observed their effectiveness and decided to implement them.

Those who registered on the central website were allowed to use the name “Death Cafe” and announce them on the site. According to the representative of the “Death Café Exchange”, some of the organizers of death cafés in Japan are registered on the central website, whereas others are not. She mentioned that the reason why many Japanese death café organizers do not register with the central website may be because they think that announcing their events on a Japanese website in the Japanese language is sufficient to promote their death café in Japan. Though organizers still respect the principles of Death Cafe, they often run their own death cafés without registering on the central website. Therefore, they do not use the name “Death Cafe” as a proper noun. Instead, they have established their own network “Death Café Exchange” to ensure quality practices, and they defined Japanese death café as a safe space for people to discuss death, offered with no intention of leading people to any conclusion, product or course of action, on a not-for-profit basis [17].

### Previous studies

There have been several studies of death cafés mainly in the USA. Miles et al. [18] reported on the spread of the concept of death café in the USA. Baldwin [19] interviewed 15 death cafés organizers and discussed the role of the death cafés facilitators as the death doulas of family communication. Hammer et al. [10] reported the potential impact of hospital-based death cafés on healthcare worker burnout. They found that 94% of hospital staff who attended these cafés believed that regular participation could have a positive effect ion burnout. Seifu et al. [14] interviewed nine death café facilitators and attendees and concluded that understanding the death café experience highlights the importance of open discussion of death, dying, and mortality in communities. In a study of death cafés outside the USA, Richards et al. [15] focused on the international death café movement. They showed competing tensions between: local translation of death café and a desire for international alignment alongside the instrumental use of death café and its incidental effects. They suggested that death cafés, while far from being a mainstream mass movement, may contribute to the de-professionalization of death and dying.

### Aim of this study

Despite the growing popularity of death cafés in Japan, there is a significant dearth of accumulated knowledge about their specifics within the Japanese context. This study was conducted by healthcare researchers who sought to gain insights into death cafés in the community, aiming to enhance death communication in clinical settings. This research focused on two key aspects to provide a comprehensive description of death cafés.

1. Underlying motivation of death café organizers: What kind of person starts a death café? What issues do they want to address by running a death café?
2. Practice of death cafés: How are death cafés organized and conducted? What are the difficulties encountered and lessons learned from organizing death cafés?

## Methods

### Study design

First, we consulted with HM, a Japanese researcher working on the Japanese death café study. Since death café participants were often one-time attendees and also highly anonymous, the organizers did not have their names or other contact information. In embarking on a study of death cafés in Japan, we decided to focus on the organizers of the death cafés.

The purpose of this qualitative descriptive study was to explore how death communication is conducted in death cafés. Through discussion within the research team members (KI, HM, TO), as a means of exploring how death communication is conducted in death cafés, an interview guide was developed to elicit the underlying motivation of organizers and the practice of death cafés in Japan. Individual in-depth interviews were performed to elicit first-hand narratives on this relatively new movement.

### Participants and sampling

The following inclusion criteria were used when inviting participants: death café organizers who 1) participated in the “Death Café Online Summit: Death Café Week 2020” organized by “Death Café Exchange” from September 22 to 27, 2020, and 2) had been running death cafés continuously for more than two years. First, HM, a researcher of Japanese death cafés who owned the ‘Death Café Online Summit: Death Café Week 2020’ directory, contacted the organizers listed in the directory. Since MH had interviewed the organizers during the ‘Death Café Online Summit: Death Café Week 2020’, a relationship was already established. MH briefly explained the purpose of the present study to the organizers and asked for permission for KI, the principal investigator, to contact them. Second, KI sent an e-mail to the organizers who gave permission to be contacted and recruited them into the study.

### Data collection

A short interview guide containing open-ended inquiries to facilitate a deep understanding of their thoughts was prepared by the research team members (Table 1). Prior to the interviews, the interviewees were provided with the interview guide, along with information on the interview’s time and location, enabling them to prepare adequately beforehand. In addition, written information about the study was sent to the participants, and any queries they may have had were addressed through Zoom or face-to-face communication, elaborating on the study’s details. After informed consent was obtained, individual semi-structured interviews were conducted according to the guide. The interviews took place between March and May 2022. Of the interviews, five were conducted face-to-face, and 11 used Zoom video conferencing software (Zoom Video Communications, Inc., San Jose, CA). Of the five face-to-face interviews, four were conducted at KI’s office, and one at the interviewee’s office. Both office spaces were conducive to a quiet and calm conversation, ensuring privacy. All interviews were conducted in Japanese by two researchers with extensive experience doing qualitative interviews: the main interviewer (KI) and the assistant interviewer (MH). The interviews lasted from 30 to 60 min.

**Table 1:** Interview guide

1	Basic information
	• About the participant: name, age, profession
	• About death cafés: first year, venue
2	Practice of death cafés
	*How do you run your death cafés? (number of staff, number of attendees, how to facilitate, topics), What makes you feel happy about running death cafés? What do you find difficult about running death cafés? What do you do to keep the death café running?*
3	Underlying motivation of death café organizers
	*What experience and thoughts led you to start a death café? What motivates you to continue running death cafés? What do you want to offer or create through death cafés?*

### Data analysis

All interviews were recorded and transcribed verbatim. Verbatim transcription was outsourced to a contractor. The resulting transcripts were de-identified by removing all identifying information from them.

In this descriptive qualitative study, the transcripts were analyzed using thematic analysis following the six-phase guide doing thematic analysis presented by Brun [20]. The six phases consist of: phase 1) familiarizing with data; phase 2) generating initial codes; phase 3) searching for themes; phase 4) reviewing themes; phase 5) defining and naming themes; and phase 6) producing the report. Common themes concerning the underlying motivation and the practice of death cafés were extracted across the transcribed interviews in an inductive manner. Initial codes were generated from the data and applied to each meaningful segment [20].

For each participant interview, this procedure was first executed by KI and TO before coming together to resolve discordances in the initial coding of all of the interviews. Following this, KI and TO worked together to group the codes by similarity, identify coherent patterns, and generate tentative themes. Lastly, these themes were reviewed by KI, TO, ST, and MH, refined according to the collated extracts, and examined for congruency with the meanings and nuances evident in the dataset as a whole.

Text fragments were coded using MAXQDA 2020 (VERBI Software). Constant comparisons between the original data and the emerging themes were conducted to maintain close connections between the data and the codes that were assigned to them. In this step of reviewing and refining themes, all authors discussed the specifics of the themes and reached a consensus.

## Results

### Characteristics of the participants

Interviews of 16 death café organizers were completed for the current study. All participants were organizers who also served as facilitators. The organizer is responsible for organizing the meeting, and the facilitator is the person who facilitates the meeting [12].

The characteristics of the participants are shown in Table 2. Of the participants, nine were male and seven were female. Three were under 30 years of age, five were in their 40 s, two in their 50 s, and six in their 60 s. Not to be personally identifiable, the participants’ professions are presented in the following categories: mental health, end-of-life care, death care, monk, and other. All participants were employed at the time of the study. Two had professional experience in end-of-life care as staff in a nursing home or hospice care facility, five were professionals in the field of mental health working as psychological counselors or nurses, and one was a professional in death care. Four were Buddhist monks, while the remaining six had a variety of other professions, such as dresser, engineer, civil servant, graphic designer, and non-profit organization employee. Three had experience volunteering in grief care or end-of-life care.

**Table 2:** Study participants and details of their death cafés

ID	Sex	Age	Profession*	First DC	Death café venue (during the pandemic)	
A	Male	50 s	Other, experience as a volunteer in grief care	2015	Buddhist temple (Buddhist temple)	
B	Male	60 s	Other, experience as a volunteer in grief care	2018	Library (Library)	
C	Female	60 s	Mental health	2015	Rental space, coffee shop (Online)	
D	Male	40 s	Monk, end-of-life care, mental health, other	2014	Library, school (Online)	
E	Female	40 s	Mental health	2018	Rental space (Online)	
F	Female	40 s	Other	2020	Buddhist temple (Online, Buddhist temple)	
G	Male	30 s	Monk	2020	Buddhist temple (Online)	
H	Female	20 s	Other	2020	Online (Online)	
I	Male	30 s	Monk	2015	Buddhist temples (Buddhist temples)	
J	Male	40 s	Monk	2018	Buddhist temple (Buddhist temple)	
K	Female	40 s	Mental health	2019	Online (Online)	
L	Male	60 s	Other, experience as a volunteer in end-of-life care	2016	Buddhist temples (Online)	
M	Female	60 s	Death care	2016	Office room (Office room)	
N	Male	60 s	End-of-life care	2017	Nursing home (Nursing home)	
O	Female	50 s	Mental health	2019	Coffee shop (Online)	
P	Male	60 s	Other	2017	Buddhist temple (Buddhist temple)	

^*^Professions are presented in the following categories: mental health, end-of-life care, death care, monk, other. *DC* death café

During the interview process, participants were not compelled to share their personal experiences of being near death. Throughout the interview, seven participants shared their personal stories naturally and spontaneously. Of them, four participants (Participants C, E, F, K) explicitly mentioned experiencing difficulty in coping with their grief, whereas two participants (Participants H, O) mentioned undergoing their own identity crises. These personal experiences ultimately motivated them to start a death café as a space to share their bereavement experiences.

Details of the death cafés that were held by the participants are shown in Table 2. The first death café was held between 2014 and 2020. Seven participants chose to hold their death cafés at Buddhist temples, whereas others chose various other venues, including libraries, rented spaces, office rooms, and nursing homes. Interestingly, two participants had already been organizing virtual death cafés prior to the COVID-19 pandemic.

### Underlying motivation of death café organizers in Japan

On the basis of thematic analysis, two main themes and six sub-themes were identified (Table 3). These themes and subthemes represent the different aspects of the underlying motivation of death café organizers. Whereas Theme A, Desire to promote personal growth and well-being, focuses on individuals, Theme B, Desire to promote community maturity and well-being, focuses on community. Community maturity, in the minds of the participants of the present study, was the transition from a death-avoiding community to a death-inclusive community.

**Table 3:** Themes and sub-themes of underlying motivation of death café organizers in Japan

A	Desire to promote personal growth and well-being	
	A1	Provide the opportunities for individuals to learn and reflect on death
	A2	Help individuals cope with the experience of bereavement
	A3	Encourage individuals to be aware of death’s inevitability and cherish life
B	Desire to promote community maturity and well-being	
	B1	Create a safe space for open conversation about death in the community
	B2	Revive the role of temples in the community
	B3	Strengthen social bonds between people

The two themes that emerged as the underlying impetus of death café organizers had contrasting orientations: individually-oriented and community-oriented. This combination of orientations was termed “individual-community orientation.” The organizers having individual orientation were consistent with the organizers (Participants C, E, F, H, K, and O) whose motives for starting death cafés were personal experiences.

–Theme A. Desire to promote personal growth and well-being.

One theme of the underlying motivation of death café organizers in Japan was to promote personal growth and well-being. Sub-themes for Theme A included:

–A1: Provide the opportunities for individuals to learn and reflect on death.

The opportunity to learn about and reflect on death is provided not only to attendees of the death café, but also to the participants themselves who organize and facilitate the sessions.

*Engaging in conversations about death provides attendees with the opportunity to gain a deeper understanding of themselves.* (Participant B)

*I hope that people gather casually at death cafés, engage in discussions, and reflect on the meaning of death and life.* (Participant I)

–A2: Help individuals cope with the experience of bereavement.

Sub-theme A2 included helping attendees develop the ability to cope not only with past bereavement but also the bereavement they will experience in the future.

*I envision death cafés as a safe and open space for care workers involved in end-of-life care, where they can freely engage in discussions and process the diverse aspects of death and dying.* (Participant N)

*One’s previous thoughts or experiences surrounding death can significantly influence how they perceive the loss of a loved one.* (Participant E)

–A3: Encourage individuals to be aware of death’s inevitability and cherish life

Subtheme A3 is the purpose of death café which was stated by its founder Crettaz [9], and is also the officially stated purpose on the central website created by Underwood [11].

*By contemplating the fact that you and your loved ones will one day pass away, you can gain clarity on what truly matters in your life. This is the type of experience that I hope to provide through death cafés.* (Participant C)

*Death cafés create an open and safe environment for attendees to discuss the reality of their own mortality, and the possibility that death could be just around the corner. This often leads to introspection on what truly matters in life and can inspire individuals to consider how they want to live moving forward.* (Participant M)

–Theme B. Desire to promote community maturity and well-being.

Another theme of the goals for running death cafés was to promote community maturity and well-being. Sub-themes for Theme B included:

–B1: Create a safe space for open conversation about death in the community.

Some participants were interested in increasing places in the community to discuss death, rather than concentrating solely on running their own death cafés.

*Death is often viewed as a taboo topic, despite its undeniable importance. I believe that the spread of death cafés and other opportunities for people to engage in open discussions about death will help to break down these barriers and encourage more meaningful conversations* (Participant D)

*The media coverage of topics such as ACP (Advanced Care Planning), euthanasia, and other death-related issues has piqued the interest of some individuals in discussing death. As a result, more people are turning to death cafés as a space to engage in these conversations. However, death cafés are not the goal. If society were more comfortable having daily conversations about death, the need for death cafés will no longer be as prevalent.* (Participant A)

–B2: Revive the role of temples in the community.

All statements categorized as B2 were made by monks.

*Temples can offer an ideal setting for hosting death cafés, as they provide a peaceful and contemplative environment that aligns well with the purpose of these gatherings. By holding death cafés at temples, we hope to create a space for ongoing dialogue about death and dying.* (Participant I)

*The topics discussed at death cafés are closely aligned with the themes that Buddhist monks explore, making temples an ideal venue for hosting these events. Through the medium of death cafés, we aim to make temples a welcoming and familiar space for individuals of all backgrounds, including those who do not necessarily identify with a particular faith* (Participant J)

–B3: Strengthen social bonds between people.

Some participants saw the death conversation not as a purpose itself, but as a tool to deepen people’s bonding.

*Death cafés provide a unique opportunity for people to engage in open and honest conversations about the deeply personal topic of death. By creating a space where individuals can connect with one another through meaningful dialogue, we hope to foster stronger bonds between people.* (Participant B)

*In today’s world, meaningful dialogue has become increasingly rare, leading to a breakdown in connections between individuals. To address this issue, I believe that fostering a culture of dialogue is essential, and death cafés can serve as a powerful way to facilitate these conversations and promote stronger bonds between people.* (Participant P)

### Practice of death cafés in Japan

Concerning the practice of death cafés, participants frequently expressed statements regarding their attitudes towards attendees with grief and their attitudes towards having both grieving and non-grieving attendees in the same session. Death café sessions, like Underwood’s Death Cafe sessions, are not intended to provide bereavement support or grief counseling [11]. However, some attendees may feel the need to process their grief. Several participants noted that involving both grieving and non-grieving attendees in the same session can pose challenges for managing the sessions.

*In settings focused on grief care, such as grief cafés, participants are often very mindful of their words and actions, as everyone present is there to support each other through the difficult process of loss. However, in contrast, death cafés operate differently, and attendees may unintentionally say things that could be hurtful to those who are currently experiencing grief.* (Participant L)

*The initial challenges I faced in running death cafés involved the situation where people who wanted to discuss the death of a loved one and those who wanted to have philosophical conversations about death were in the same session.* (Participant D)

Some participants expressed strong emotional tension about the situation of having grieving and non-grieving attendees in the same session and tried to avoid such a situation from taking place in their death cafés. On the other hand, some participants stated that they were not too concerned about such a situation and emphasized that their death cafés were open to any attendees. Focusing on the participants’ attitude towards having attendees with and without grief in the same session, the practice of death cafés in Japan was categorized into two categories (Table 4). Two themes emerged as the practices of death cafés had contrasting orientations: inclusive and purification. This combination of orientations was termed “purification-inclusive orientation.”

**Table 4:** Categories and sub-categories of the practice of death cafés in Japan

a	Inclusive	
	a1	Run death cafés targeting grieving attendees and non-grieving attendees
b	Purification	
	b1	Run death cafés targeting grieving attendees
	b2	Run death cafés targeting non-grieving attendees

-Category a: Inclusive.

The majority of the participants thought that, in death cafés, it was inevitable that grieving and non-grieving participants would be in the same session. We call this attitude “Inclusive”. They acknowledge that grieving attendees and non-grieving attendees may have distinct needs, but they believe that a death café provides a space where people with diverse needs can come together.

*I occasionally receive feedback from attendees who express a desire for me to have listened to them more closely regarding their grief. However, I recognize that a death café is not the ideal setting for such personalized attention, so I do not dwell on these comments. If I were to allow myself to be overly concerned with every individual’s feedback, I would not be able to continue running these events.* (Participant M)

*When I started running death cafés, I was expecting it to be very difficult to manage sessions with grieving and non-grieving attendees together. However, in reality, it went well. I believe that the unique features of death cafés, where people of different ages, positions, values, and motivations for attending the gathering, and most of them meet just once, have a positive impact on the gathering. (Participant J)*

-Category b:* Purification*

Some participants (Participants C, E, K, and O) avoided having grieving attendees and non-grieving attendees in the same session. Participants E and K ran death cafés targeting grieving attendees, whereas Participants C and O recommended non-death café settings, such as grief cafés or individual counseling sessions. Their death cafés targeted non-grieving attendees. The attitudes that seek to avoid having grieving attendees and non-grieving attendees in the same session were labeled as “purification”.

*Many of my death café attendees are grieving and may not be ready for professional grief counseling. I welcome them and create a safe space for them to openly discuss their feelings and experiences as they learn to confront their grief.* (Participant K)

*If someone is currently grieving, I believe that joining a death café may result in an atmosphere that differs from my intended vision for a death café. Therefore, I highly recommend that they attend my grief cafés.* (Participant O)

Additionally, Participants F and H stated that grieving attendees do not come to their death cafés. They mentioned that this may be because the announcement statement conveys that their death cafés are not suitable for grieving people.

*When recruiting attendees for my death cafés, I am upfront about my use of graphic recordings and my intention to upload them to social networking sites. Individuals with grief may not feel that my death café is the right fit for them, and as a result, rarely attend.* (Participant H)

The participants intentionally avoiding or unintentionally not having grieving attendees and non-grieving attendees in the same session matched with those who disclosed their bereavement experience, which served as the motive for starting a death café.

### Classification of Japanese death cafés

**Fig. 1:**
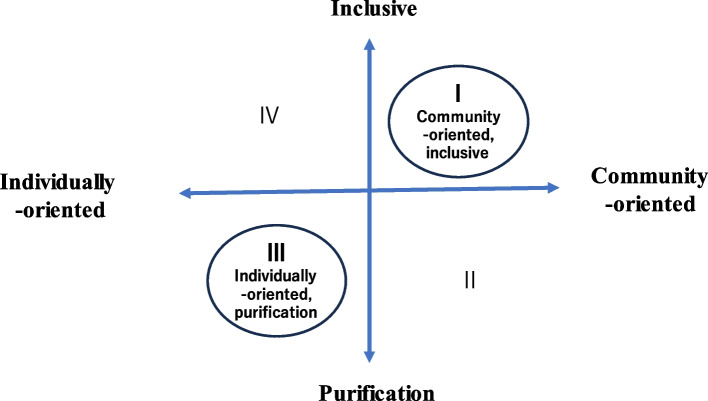
Classification of death cafés in Japan

To gain insight into the dynamics of Japanese death cafés, a matrix was developed, aligning underlying motivation (individually oriented/community oriented) on the horizontal axis and the practice of death cafés (purification/inclusive of grieving and non-grieving attendees) on the vertical axis (Fig. 1). Japanese death cafés were thus categorized into quadrants 1 (community oriented, inclusive) and 3 (individually oriented, purification), whereas quadrants 2 and 4 remained vacant. Notably, the death cafés in quadrant 1 were frequently held in temples.

## Discussion

Examining the underlying motivation of death café organizers in Japan, two themes, i.e. “desire to promote personal growth and well-being”, which focuses on individuals, and “desire to promote community maturity and well-being”, which focuses on community, emerged, with contrasting orientations, which was termed “individually-community orientation.” Examining the practice of death cafés in Japan, the focus was on the “attitude towards having grieving and non-grieving attendees in the same death café session.” Participants’ attitudes towards this situation fell into two categories with opposing orientations: “purification-inclusion orientation.”

When a matrix was developed aligning underlying impetus (individually oriented/community oriented) on the horizontal axis and the practice of death cafés (purification/inclusive of grieving and non-grieving attendees) on the vertical axis, all of the death cafés in the present study were categorized into quadrants 1 (community oriented, inclusive) and 3 (individually oriented, purification), with quadrants 2 and 4 remaining vacant. Furthermore, the death cafés in quadrant 1 often took place in temples.

Considering that the purification orientation was more prevalent among organizers who had their personal experiences as a motive for starting the death café, the universal application of this type of death café in clinical settings is not realistic. Furthermore, since what is needed in a clinical setting is a move towards a community where people can openly discuss death, the application of the community-oriented type is recommended. Therefore, community-oriented and inclusive death cafés, many of which are held in temples, are key.

It was not our initial assumption that temples play a significant role in death communication. However, this finding is rational considering that Buddhism has been addressing death in Japan for 1500 years, whereas the history of death communication in modern Japanese clinical settings is relatively short. During ancient and medieval times, Chinese and Japanese monks wrote books on death practices, greatly influencing the modern and early modern periods and shaping the Japanese attitude toward death and dying [21]. On the other hand, it was not until 1981 that the first hospital with a care program for the dying in Japan, a hospice, was established. Considering that before the 1980s, people were reluctant to discuss death in hospitals, the finding that temples play a significant role in death communication becomes more reasonable.

Reports have been published on the practice of caregiver cafes in temples [22]. With approximately 77,000 Buddhist temples in Japan, temples have the potential to serve as valuable social resources for death cafés. Participants noted that “temples are safe and welcoming spaces that encourage self-disclosure” (Participant L), and “Attendees say they have the impression that temples are safe places where they can speak freely without fear of judgment or harm” (Participant J). The factor of a temple as a safe place for self-disclosure contributes to the suitability of temples as venues for death cafés, which aim to provide a supportive and non-judgmental environment for discussing topics related to death and dying.

According to a participating monk in our study, monks hold a neutral stance towards death: “Monks are in a position of neither denying nor affirming death. We simply recognize it as something that is inevitable for people. Monks may be well-suited to facilitate death cafés due to our neutral stance towards death” (Participant I). However, another monk’s personal experience with his father’s death showed that the reality of death can be quite different from one’s previous understanding of it: “After my father’s death, I realized that the reality of death was quite different from what I had previously understood as a monk” (Participant D).

During the interviews, several participants noted that the practices of death cafés reflect the organizer’s views on death and dying. Hammer et al. [10], who conducted a study of hospital-based death cafés, assumed that death was considered a burden and that staff engaged in end-of-life care in hospitals were at risk of burnout, and the cafés were intended to address this. In Japan, the discussion of “good death,” which began in the area of cancer palliative care [23], is expanding to cover a wider range of diseases [24, 25]. However, it is also true that healthcare professionals see death and dying as a burden [26]. Further communication between healthcare professionals and temple death café organizers is recommended.

## Conclusions

The underlying motivation of death café organizers in Japan was either individually oriented or community oriented. Similarly, the practices of death cafés in Japan were either purification-focused or inclusive. Japanese death cafés can be categorized as either individually and purification-oriented or community and inclusive-oriented. As healthcare professionals, there is indeed much to be learned from death café organizers, particularly in the realm of promoting death communication. Specifically, temple death cafés, with their inclusive practices and orientation toward community, can be particularly helpful in fostering a sense of inclusivity and community engagement.

### Limitation and further research recommendation

The first limitation concerns the participants. The attendees of the death cafés were not included. Accordingly, the effect of death cafés on attendees was not explored. Research on attendees is a further challenge.

The second limitation concerns the methods. The data were retrospective, organizer-based data. The real climate of the death cafés was not explored. Participant observation research is also a further challenge.

The third limitation concerns the methodology. How the death cafés were run was described, but the mechanism remains unclear. Conducting in-depth interviews with attendees and performing phenomenological analyses will help us gain better understanding of the mechanism.

## Data Availability

The datasets generated and analyzed during the current study are not publicly available due to the need to protect participants’ confidentiality but they are available from the corresponding author on reasonable request.

## Abbreviation

ACP: Advance care planning
